# The changes of electroencephalography in mountaineers on Mount Jade, Taiwan: An observational study

**DOI:** 10.1371/journal.pone.0275870

**Published:** 2022-11-23

**Authors:** Kuo-Song Chang, Yu-Hui Chiu, Wei-Fong Kao, Cheryl C. H. Yang, Chorng-Kuang How, Yen-Kuang Lin, Yuh-Shyan Hwang, Ding-Kuo Chien, Ming-Kun Huang, Terry B. J. Kuo

**Affiliations:** 1 Department of Emergency Medicine, MacKay Memorial Hospital, Taipei, Taiwan; 2 Department of Medicine, MacKay Medical College, New Taipei City, Taiwan; 3 MacKay Junior College of Medicine, Nursing, and Management, Taipei, Taiwan; 4 Department of Emergency, School of Medicine, College of Medicine, Taipei Medical University, Taipei, Taiwan; 5 Department of Emergency and Critical Care Medicine, Taipei Medical University Hospital, Taipei, Taiwan; 6 Institute of Brain Science, National Yang Ming Chiao Tung University, Taipei, Taiwan; 7 Sleep Research Center, National Yang Ming Chiao Tung University, Taipei, Taiwan; 8 Brain Research Center, National Yang Ming Chiao Tung University, Taipei, Taiwan; 9 Department of Education and Research, Taipei City Hospital, Taipei, Taiwan; 10 Department of Emergency Medicine, Taipei Veterans General Hospital, Taipei, Taiwan; 11 Department of Emergency Medicine, School of Medicine, National Yang Ming Chiao Tung University, Taipei, Taiwan; 12 Graduate Institute of Athletics and Coaching Science, National Taiwan Sport University, Taoyuan, Taiwan; 13 Department of Electronic Engineering, National Taipei University of Technology, Taipei, Taiwan; 14 Clinical Research Center, Taoyuan Psychiatric Center, Ministry of Health and Welfare, Taoyuan, Taiwan; UNITED KINGDOM

## Abstract

**Background:**

The diagnosis of acute mountain sickness, which lacks a reliable and objective diagnostic tool, still depends on the clinical symptoms and signs and remains a major threat and unpredictable disease affecting millions of mountaineers.

**Objectives:**

To record electroencephalography signals with small, convenient, wireless equipment and to test whether electroencephalography parameters, which are more sensitive and reliable markers, could predict the symptoms of acute mountain sickness.

**Methods:**

Twenty-five participants were enrolled and separated into two groups to climb Mount Jade in Taiwan. We collected electrocardiography signals and arterial oxygen saturation data at ground, moderate (2,400 m), and high altitude (3,400 m). A spectral analysis of the electrocardiography was performed to assess the study subjects’ electroencephalography activity at different frequencies (α, β, θ, δ) and the mean power frequency of electrocardiography. The clinical symptoms and Lake Louise Acute Mountain Sickness scores of the subjects were recorded for comparison.

**Results:**

A significant change in the δ power of electroencephalography was recorded in subjects ascending from the ground to a high altitude of 3,400 m in a 4-day itinerary. In addition, between the two groups of subjects with and without acute mountain sickness (Lake Louise Acute Mountain Sickness scores < 3 and ≥ 3), the δ power of electroencephalography at the fronto-parietal 1 and parietal 3 electrodes at moderate altitude as well as the changes of δ power and mean power frequency of electrocardiography over parietal 4 at high altitude showed a significant difference. At moderate altitude, the increasing δ power of electroencephalography at the parietal 4 electrode was related to the headache symptom of acute mountain sickness before ascending to high altitude.

**Conclusion:**

At moderate altitude, the δ power increase of electroencephalography at the P4 electrode could be a predictor of acute mountain sickness symptoms before ascending to high altitude. Thus, electroencephalography had the potential to identify the risk of acute mountain sickness.

## Introduction

Acute mountain sickness (AMS) is a non-specific syndrome characterized by the presence of headaches and at least one of the following symptoms when people arrive at an altitude above 2,500 m: loss of appetite, nausea, vomiting, fatigue/weakness, dizziness/light-headedness, or insomnia [1]. The most important risk factors for the development of high-altitude illness are the rate of ascent, altitude reached (especially sleeping altitude), and individual susceptibility [1, 2]. Although the exact AMS-inducing mechanism remains unknown, a close link between the altered adaptation of the central nervous system to the brain and the control of peripheral cardiovascular functions during hypoxia at high altitude has been hypothesized [3]. Without treatment, AMS can evolve into high-altitude cerebral edema (HACE) and high-altitude pulmonary edema (HAPE), which are defined as disturbances of consciousness, and acute interstitial edema of the lung, which may progress to deep coma, confusion, ataxia, dyspnea, and respiratory failure. HACE and HAPE cause significant morbidity and occasionally death in otherwise perfectly healthy persons [4].

AMS is the most common problem, affecting 25% of people at altitudes of 1,850–2,750 m [5], 42% at altitudes of 3,000 m [6], and as many as 75% of those attempting to climb Mt. Kilimanjaro (5,984 m) [7]. The rapid and proper recognition of symptoms of AMS is key to avoiding disease deterioration to HACE and HAPE and diminishing morbidity. Although the arterial oxygen saturation (SPO_2_) is useful in predicting AMS [8–10], the use of pulse oximetry under field conditions is susceptible to many disruptive factors [11]. Currently, no objective marker exists for AMS diagnosis, and no predictive tool is available to determine whether subjects are prone to developing symptoms of AMS at high altitudes. Millions of mountaineers and tourists can only depend on their experience and clinical symptoms/signs to identify and prevent symptoms of AMS when exposed to hypobaric hypoxic environments at high altitudes [1, 12].

A non-invasive, specific, reliable, and convenient method under field conditions is needed to detect inadequate acclimatization and impending AMS. Several studies have found an increase of slow cerebral activity in the right temporal region [13, 14]. Understanding the sequential changes in electroencephalography (EEG) in the brain during progressive exposure to hypobaric hypoxia while ascending from lower to higher altitudes may provide invaluable information for the prediction of AMS at high altitudes [15, 16]. Under application with small, convenient, wireless, non-invasive polysomnographic recording equipment, which can collect EEG signals, these EEG parameters can be more easily approached and popularized among non-professional mountaineers. On the basis of a similar technique and methodology of previously reported articles, this study was designed to determine the correlation between parameters of EEG and symptoms of AMS and to compare which parameter at moderate altitude is a more specific and reliable tool for the prediction of more severe symptoms of AMS at high altitude.

## Materials and methods

### Participants

This prospective observational study included 25 participants who joined itineraries separately in June and November 2010. All the subjects lived at an altitude of less than 500 m above sea level, and none of them had been exposed to an altitude above 2,000 m in the 4 months prior to this study. None of the patients had medical problems, such as hypertension, diabetic neuropathy, or cardiovascular and pulmonary diseases, which could have affected the analysis and interpretation of the EEG.

### Ethics statement

All subjects provide written informed constant approved by the Institutional Review Board of Taipei Veterans General Hospital (97-10-08A).

### Procedure

This study was designed as a 4-day itinerary by trekking to Mount Jade (3,952 m) in Taiwan. The route began from the ground (Taipei city) to Lulin lodge (2,400 m) by taking a bus on the first day, trekking to Paiyun lodge (3,400 m) on the second day, climbing Mt. Jade on the third day, and going down to sea level on the fourth day. Basic data, EEG signals, SpO_2_, and the Lake Louise Acute Mountain Sickness (LLAMS) scores were recorded on the day before the itinerary in Taipei (sea level), the first day in Lulin lodge at moderate altitude, and the second day in Paiyun lodge (high altitude). Considering the load of trekking from 2,400 m to 3,400 m, we collected these parameters after more than 1 hour of rest in Paiyun lodge on the second day. All data were obtained from approximately 4 pm to 6 pm in the evening under rest conditions before the meal.

### Measurements

#### Pulse oximeter

SpO_2_ and pulse rate (PR) were recorded using an NPB-40 handheld pulse oximeter (Nellcor Puritan Bennett Inc., Pleasanton, California). The SpO_2_ probe was set on the right index finger while the participants quietly sat on a chair and rested for at least 5 minutes.

#### Polysomnographic recording

**Fig 1:**
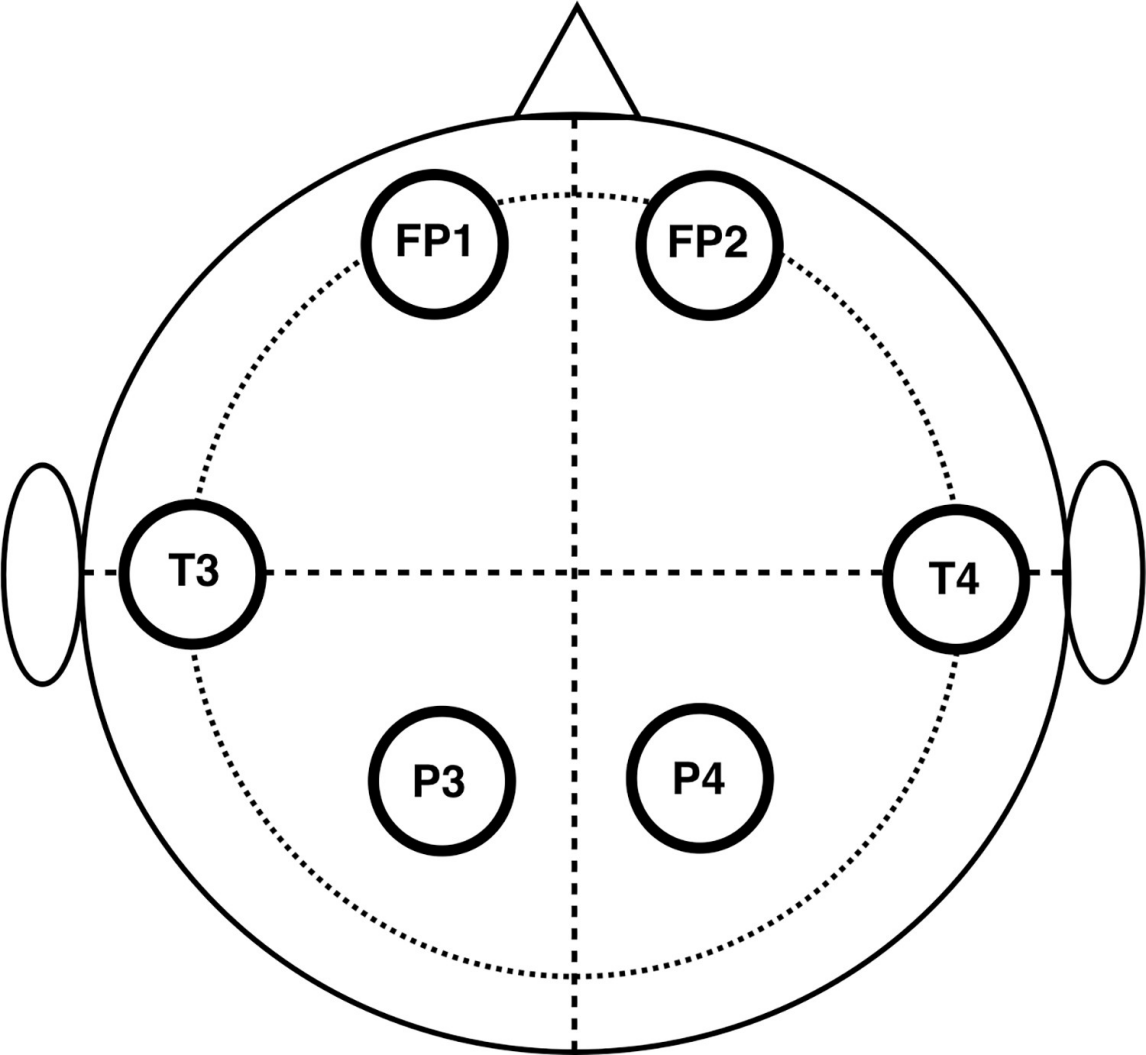
The position of electroencephalography signals recording, using 10–20 systems. FP1 & FP2, fronto-parietal electrode 1 & 2; T3 & T4, temporal electrode 3 & 4; P3 & P4, parietal electrode 3 & 4.

Data recording of all subjects was performed in the evening following more than 1 hour of rest after trekking to the scheduled destinations. EEG signals were recorded using wireless polysomonographic recordings (TD1; Taiwan Telemedicine Device Company, Taiwan). Detailed procedures for the EEG have been described in previous articles [17–19]. EEG signals at the frontoparietal 1 (FP1), frontoparietal 2 (FP2), temporal 3 (T3), temporal 4 (T4), parietal 3 (P3), and parietal 4 (P4) electrodes were recorded for 3.5 minutes with silver electrodes and placed according to 10–20 systems, while subjects sat quietly with their eyes closed (Fig 1).

#### Lake Louise Acute Mountain Sickness scores

The LLAMS, a well-validated standard [20], was used to evaluate the symptoms of AMS. The subjects presented the following symptoms: headache, gastrointestinal upset (loss of appetite, nausea, and vomiting), fatigue/weakness, or dizziness/light-headedness; a LLAMS score ≥ 3 indicated symptomatic AMS. All subjective symptoms and objective tests were examined and recorded by experienced emergency physicians.

#### Electroencephalography acquisition and analysis

Electrophysiological signals were recorded using a miniature physiological signal recorder (TD1; [17–19]. The small size (5.2 × 3.1 × 1.2 cm^3^) and light weight (11 g) of the recorder produced a minimum interference on the participants. The EEG signals were recorded using an analog-to-digital converter with a sampling rate of 256 Hz. All data were stored on a memory card inside the equipment and processed on an HP-compatible portable personal computer.

In this study, we recorded EEG signals over six electrodes for 3.5 minutes. From each 210-s EEG signal, we selected three 32-s epochs with the least interference and noise manually for data analysis. The EEG data file was processed using a special computer program in Pascal language (Borland Pascal 7.0, Borland, USA) for bioelectric signals [21]. The digital signal processing of the bioelectric signals was similar to that adopted in our previous studies [18, 19]. We used a 32-s window length to provide a frequency resolution of 0.5 Hz. The EEG was resampled at 64 Hz and truncated into successive 32-s time segments (windows or epochs), and a Hamming window was applied to each time segment to attenuate the leakage effect. Our algorithm then estimated the power density of the spectral components based on fast Fourier transformation (FFT). The resulting power spectrum was corrected for attenuation resulting from the sampling and application of the Hamming window. For each time segment, we calculated the mean power frequency of EEG (MPF_EEG_) and amplitude power of EEG within the δ, θ, β, and α ranges (total power, 0.5–32 Hz, 0.5–4 Hz, 4–8 Hz, 16–32 Hz, 9–11 Hz).

#### Statistical analysis

The Shapiro–Wilk test (p > 0.05) and an inspection of the participants’ histograms, normal Q-Q plots, and box plots were used to test the normality of our data. Descriptive results were reported as mean ± SD. We applied a repeated measures analysis of variance (RM-ANOVA; or Friedman test when appropriate) to evaluate the association between the three altitudes (ground, 2,400 m, and 3,400 m). The numerical parameters of the EEG power spectrum at 2,400 m and 3,400 m altitudes were compared to the baseline values at the ground level using a paired samples t-test (or Wilcoxon signed ranks test when appropriate). The Mann-Whitney U test was used to evaluate the association between subjects with and without AMS, with and without headache, and with LLAMS < 5 and LLAMS ≥ 5. Significant differences were found using the least significant difference (LSD) as post-hoc test after adjustment for multiple comparisons. The correlation between EEG power spectrum changes and LLAMS scores was calculated using the Pearson’s correlation coefficient. Commercially available statistical software (SPSS version 21.0, IBM Corp, Armonk, NY) was used for statistical analysis. Differences were considered to be statistically significant when 2 tailed *p* < 0.05.

## Results

### Characteristics of study subjects

This study included 25 non-smoking participants (mean ± SD, 32.4 ± 9.2 years; 10 males, and 15 females). Only one participant presented more than three symptoms of AMS at 2,400 m, and 11 of the 25 subjects were diagnosed with AMS (LLAMS ≥ 3, n = 11) at 3,400 m. The prevalence of acute mountain sickness was 44%. Among these 11 participants, two were male, and nine were female (Table 1). No statistical significance in age, sex, body mass index and sleep quality at 2,400 m and 3,400 m between subjects with (LLAMS ≥ 3) and without AMS was noted.

**Table 1:** Basic characteristics and LLAMS score among all participants, without (LLAMS < 3) and with AMS (LLAMS ≥ 3) groups.

Characteristics	All (n = 25)	LLAMS < 3 (n = 14)	LLAMS ≥ 3 (n = 11)	LLAMS < 3 vs ≥ 3 (P-value)
Age, years	32.36 ± 9.22	31.36 ± 7.74	33.63 ± 10.67	0.647
Sex, male/female	10/15	8/6	2/9	0.099
Smoker/non-smoker	0/25	0/14	0/11	1.000
BMI, kg/m^2^	20.58 ± 2.74	20.78 ± 2.86	20.33 ± 2.70	0.694
Sleeping quality at 2,400 m				0.151
Sleep as well as usual	15	7	8	
Did not sleep as well as usual	9	7	2	
Woke many times, poor night’s sleep	1	0	1	
Sleeping quality at 3,400 m				0.757
Sleep as well as usual	4	3	1	
Did not sleep as well as usual	11	6	5	
Woke many times, poor night’s sleep	10	5	5	
LLAMS at 2,400 m	0.68 ± 0.46	0.64 ± 0.71	0.73 ± 0.96	-
LLAMS at 3,400 m	3.00 ± 1.96	1.57 ± 0.62	4.82 ± 1.53	-

Values presented as mean ± SD; LLAMS = Lake Louise Acute Mountain Sickness; AMS = acute mountain sickness; vs = versus; BMI = body mass index.

### Comparison of SPO_2,_ PR, and power spectrum of EEG at different altitudes

Table 2 shows the comparison of SPO_2,_ PR, and the power spectrum of EEG at different electrodes among different altitudes. At moderate altitude (2,400 m), SPO_2_, PR, MPF_EEG_ at P4, and ratio of EEG power at P3 had significant changes (*p* < 0.05) when compared to the values at the ground level. At 3,400 m, SPO_2_, PR, MPF_EEG_ at P3, and ratio of EEG at P3 had significant changes (*p* < 0.05) when compared to the values at the ground level. A comparison of the parameters between altitudes (at 2,400 m and 3,400 m) revealed a statistically significant change (*p* < 0.05) in SPO_2_, PR, MPF_EEG_ at P3 and P4, δ power at FP1, and ratio of EEG power at P3. Significant changes with the increase of slow EEG were observed in subjects traveling from ground to high altitude.

**Table 2:** SpO_2,_ PR, and EEG parameters comparison between different altitudes at ground, 2,400 m, and 3,400 m.

		Ground	2,400 m	3,400 m			Ground	2,400 m	3,400 m
	SPO_2_^||^ (%)	98.43 ± 1.14	92.96 ± 4.17^†^	84.76 ± 5.22^†※^		PR^||^ (bpm)	73.13 ± 9.74	76.76 ± 11.69*	102.48 ± 13.99*^#^
MPF_EEG_ (Hz)	FP1	6.86 ± 1.96	6.59 ± 1.78	6.83 ± 1.78	Ratio [ln(ratio)]	FP1	0.76 ± 0.16	0.75 ± 0.16	0.76 ± 0.16
	FP2	6.87 ± 1.67	6.49 ± 1.89	6.51 ± 1.51		FP2	0.73 ± 0.16	0.72 ± 0.16	0.73 ± 0.14
	T3^||^	9.69 ± 1.56	9.25 ± 2.11	10.33 ± 1.74		T3	0.99 ± 0.16	0.94 ± 0.19	1.03 ± 0.17
	T4	9.38 ± 1.93	9.49 ± 1.62	10.31 ± 2.42		T4	0.96 ± 0.22	0.97 ± 0.21	1.03 ± 0.21
	P3^||^	9.65 ± 0.96	9.15 ± 1.34	10.01 ± 1.20*^#^		P3	1.20 ± 0.20	1.11 ± 0.32^†^	1.31 ± 0.20*^#^
	P4	9.85 ± 0.85	9.19 ± 1.42^†^	9.84 ± 1.31^#^		P4	1.20 ± 0.21	1.14 ± 0.31	1.24 ± 0.28
δ [ln(uV^2^)]	FP1^||^	4.45 ± 1.05	4.67 ± 0.87	4.32 ± 0.79^※^	θ [ln(uV^2^)]	FP1^||^	2.64 ± 0.68	2.84 ± 0.84	2.72 ± 0.69
	FP2^||^	4.28 ± 0.89	4.60 ± 0.82	4.47 ± 0.74		FP2^||^	2.76 ± 0.64	2.78 ± 0.73	2.66 ± 0.63
	T3^||^	3.35 ± 0.75	3.46 ± 0.71	3.31 ± 0.51		T3^||^	2.38 ± 0.49	2.60 ± 0.63	2.49 ± 0.57
	T4^||^	3.43 ± 0.84	3.27 ± 0.62	3.34 ± 0.63		T4	2.50 ± 0.53	2.38 ± 0.45	2.53 ± 0.63
	P3^||^	2.83 ± 0.59	2.99 ± 1.10	2.67 ± 0.61		P3	2.29 ± 0.67	2.40 ± 1.08	2.11 ± 0.81
	P4^||^	2.83 ± 0.59	2.93 ± 0.70	2.68 ± 0.70		P4	2.33 ± 0.60	2.29 ± 0.68	2.18 ± 0.69
α [ln(uV^2^)]	FP1	2.97 ± 0.71	3.06 ± 0.78	2.89 ± 0.64	β [ln(uV^2^)]	FP1^||^	2.34 ± 0.65	2.58 ± 0.91	2.38 ± 0.56
	FP2	2.84 ± 0.85	2.85 ± 0.73	2.76 ± 0.60		FP2^||^	2.29 ± 0.62	2.40 ± 0.79	2.37 ± 0.52
	T3	2.98 ± 0.57	2.96 ± 0.62	3.02 ± 0.69		T3^||^	2.62 ± 0.59	2.65 ± 0.67	2.90 ± 0.78
	T4	2.95 ± 0.65	2.84 ± 0.57	3.05 ± 0.59		T4	2.62 ± 0.67	2.56 ± 0.58	2.91 ± 0.90
	P3	3.64 ± 0.92	3.44 ± 1.16	3.48 ± 1.06		P3	2.39 ± 0.70	2.28 ± 0.95	2.36 ± 0.74
	P4	3.69 ± 1.16	3.51 ± 1.06	3.32 ± 0.89		P4	2.46 ± 0.58	2.24 ± 0.66	2.50 ± 0.98

Values presented as mean ± SD; SPO2, arterial blood oxygen saturation; PR, pulse rate; EEG, electroencephalography; bpm, beats per minute; MPF_EEG_, mean power frequency of EEG; Ratio, electroencephalography power spectrum radio (α+β) / (θ+δ); FP1 & FP2, fronto-parietal electrode 1 & 2; T3 & T4, temporal electrode 3 & 4; P3 & P4, parietal electrode 3 & 4.

* *p* < 0.05, significantly higher than the value at ground

†*p* < 0.05, significantly lower than the value at ground

# *p* < 0.05, significantly higher than the value at 2,400 m

^※^*p* < 0.05, significantly lower than the value at 2,400 m

^||^p-value was obtained using the Wilcoxon signed ranks test.

### Parameters of EEG between two groups with and without AMS

**Fig 2:**
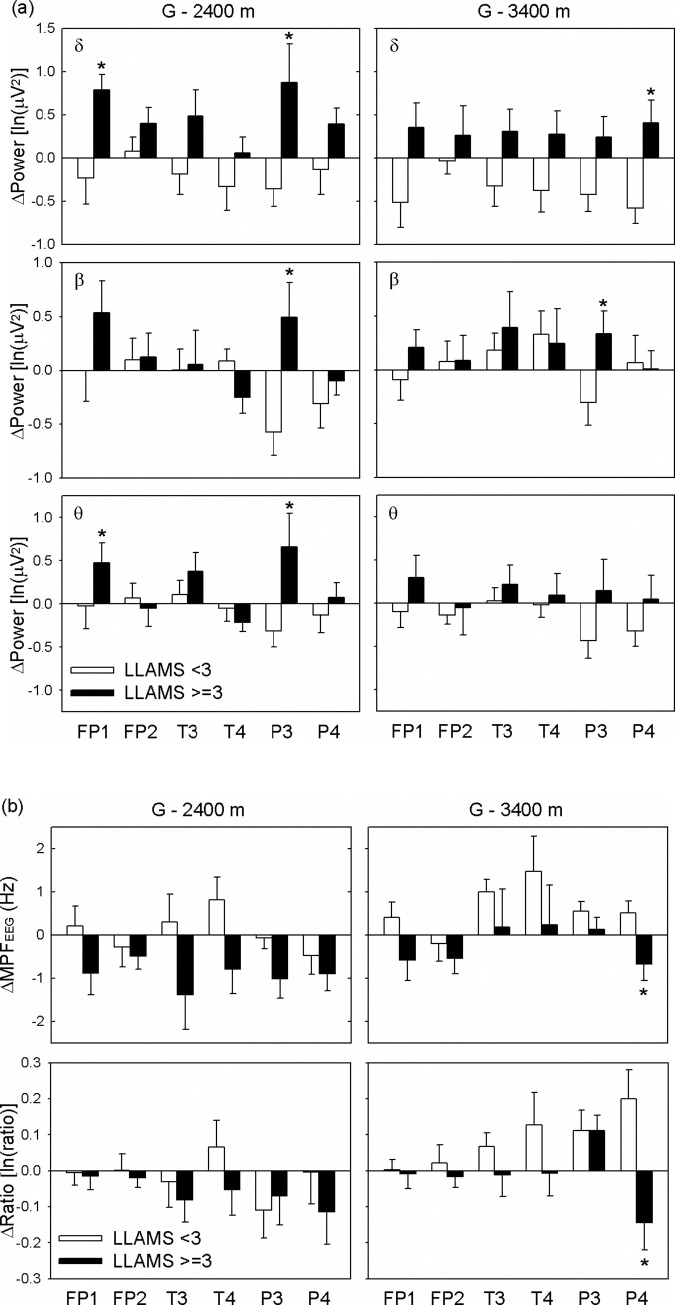
Electroencephalography power spectra (A) and ratio and mean power frequency of electroencephalography (B) at different electrodes, and the relationship between two groups of subjects with and without AMS from the ground to 2,400 m and 3,400 m. The results are presented as estimated marginal mean ± standard error (SE). *p < 0.05, compared with the asymptomatic group (LLAMS < 3). Without AMS group (LLAMS < 3, n = 14); with AMS group (LLAMS ≥ 3, n = 11). AMS, acute mountain sickness; LLAMS, Lake Louise Acute Mountain Sickness scores; FP1 &FP2, fronto-parietal electrode 1 & 2; T3 & T4, temporal electrode 3 & 4; P3 & P4, Parietal electrode 3 & 4; MPF_EEG_, mean power frequency of electroencephalography; Ratio, electroencephalography power spectrum radio (α+β) / (θ+δ).

All symptomatic subjects had normal EEG parameters at baseline and a relatively increased frequency power of EEG with increasing altitude. The EEG frequency power at different electrodes of the two groups was compared. From the ground to 2,400 m, the Δδ power of EEG at FP1 and P3, the Δβ power of EEG at P3, and the Δθ power of EEG at FP1 and P3 showed statistical significance between the two groups of LLAMS < 3 and LLAMS ≥ 3 at 2,400 m (Fig 2A). From the ground to 3,400 m, changes in the Δδ power of EEG at P4, Δβ power of EEG at P3, ΔMPF_EEG_ and Δratio of EEG at P4 were statistically significant between the non-AMS and AMS groups (Fig 2A and 2B).

### Analysis of EEG parameters among subjects with headache, moderate AMS severity, and correlation of LLAMS scores

**Fig 3:**
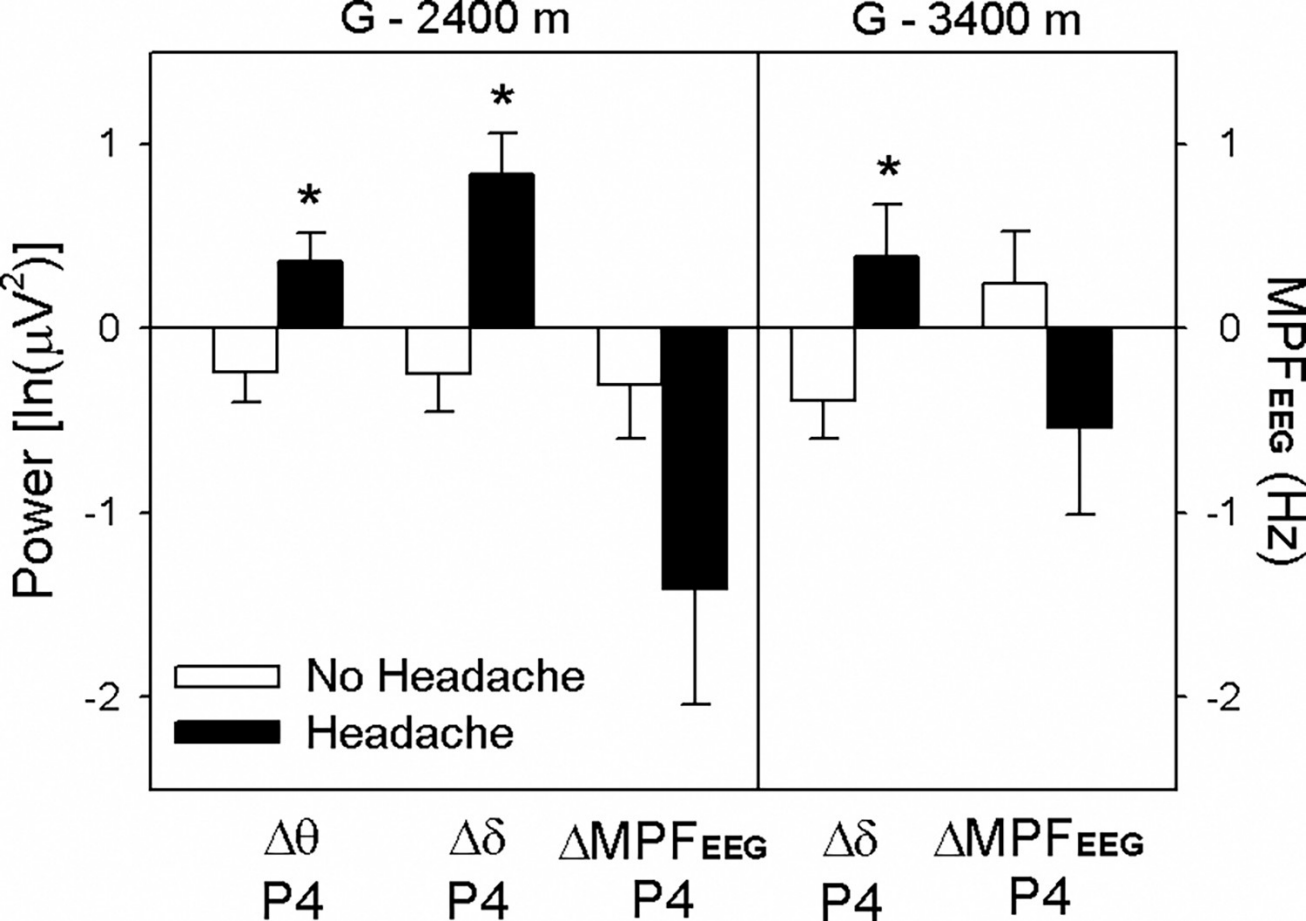
Comparison of the difference in θ, δ power spectral activity and mean power frequency of electroencephalography at the P4 electrode from ground to 2,400 m and to 3,400 m between two groups without and with the headache symptom. The results are presented as estimated marginal means ± standard errors (SE). *p < 0.05, compared with the non-headache group. Without headache group (n = 17); with headache group (n = 8). MPF_EEG,_ mean power frequency of electroencephalography; P4, parietal electrode 4.

**Fig 4:**
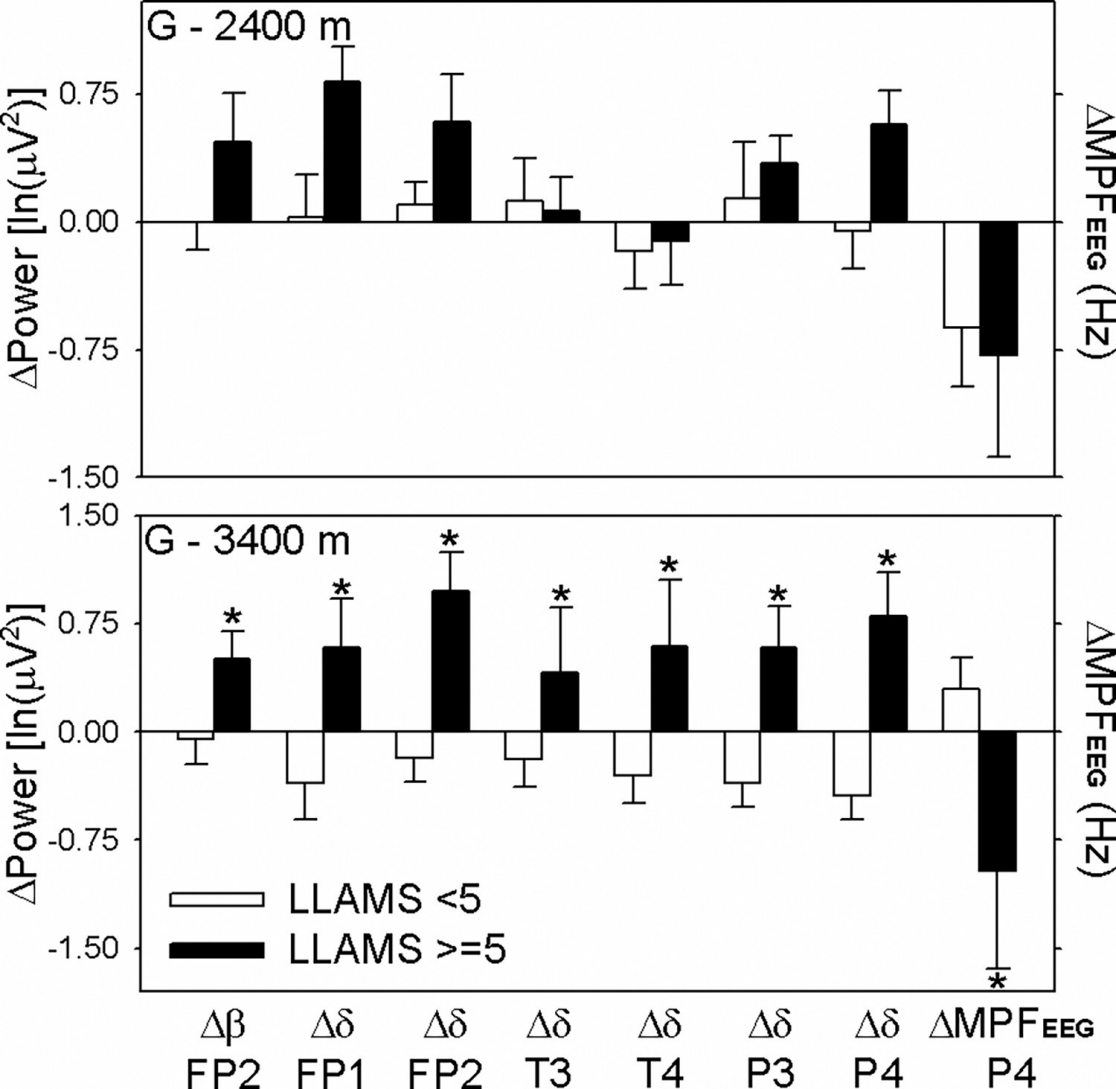
Changes of electroencephalography power spectrum over different electrodes from ground to 2,400 m and to 3,400 m between two groups with moderate and minor AMS severity. The results are presented as estimated marginal means ± standard errors (SE). *p < 0.05, compared with the minor AMS severity group (LLAMS < 5). With minor AMS group (LLAMS < 5, n = 19); With moderate AMS group (LLAMS ≥ 5, n = 6); AMS: acute mountain sickness; LLAMS, Lake Louise Acute Mountain Sickness scores; MPF_EEG,_ mean power frequency of electroencephalography; FP1 & FP2: fronto-parietal electrode 1 & 2; T3 & T4: temporal electrode 3 & 4; P3 & P4: parietal electrode 3 & 4.

**Fig 5:**
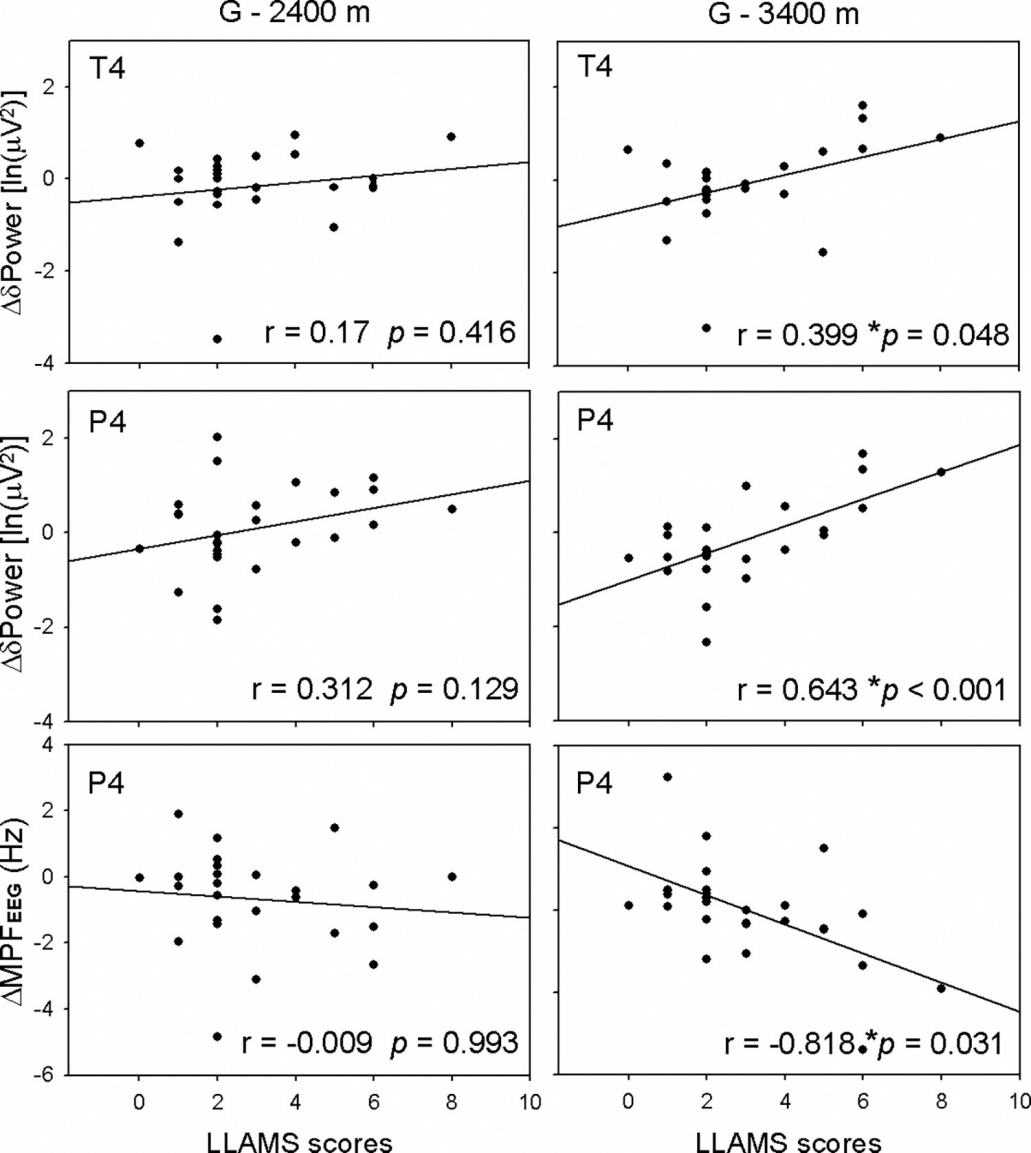
The differences of δ power spectrum at T4 and P4 and of MPF_EEG_ at the P4 electrode from ground to 3,400 m were significantly corrected with LLAMS scores, but not from ground to 2,400 m. Correlation between slow cerebral activity and the severity of LLAMS scores. Significant differences were found using the least significant difference (LSD) as *p < 0.05 by Pearson’s correlation. LLAMS, Lake Louise Acute Mountain Sickness; MPF_EEG,_ mean power frequency of electroencephalography; T4, temporal electrode 4; P4, parietal electrode 4.

As headache is an important symptom of acute mountain sickness and is an essential criterion for AMS diagnosis, we compared EEG parameters with and without the symptom of headache at 3,400 m (Fig 3). The Δθ and Δδ power at the P4 electrode significantly increased from the ground to 2,400 m. The Δδ power at the P4 electrode significantly increased from the ground to 3,400 m. However, the decrease of ΔMPF_EEG_ from the ground to both 2,400 m and 3,400 m had no statistical significance. When we compared the moderate severity of AMS as LLAMS ≥ 5 with minor symptoms (LLAMS < 5), we observed significant differences in EEG parameters from the ground to 3400 m (Fig 4). The difference from the ground to 3,400 m of Δβ power at FP2, Δδ power at all six electrodes, and ΔMPF_EEG_ at P4 had statistical significance between the LLAMS < 5 and LLAMS ≥ 5 groups. It revealed a more prominent increase in Δδ power in people with more symptoms of AMS (LLAMS ≥ 5) than in people with fewer symptoms (LLAMS < 5). These increases in Δδ power from the ground to 3,400 m at the T4 and P4 electrodes were correlated with LLAMS scores (r = 0.399, *p* = 0.048; r = 0.643, *p* < 0.001; Fig 5). The decrease in ΔMPF_EEG_ was also correlated with LLAMS scores (r = -0.818, *p* = 0.031).

## Discussion

In this study, we demonstrated a significant change in the δ power of EEG in healthy participants ascending from the ground to a high altitude of 3,400 m in a 4-day itinerary. Although changes in δ power are considered to be associated with sleep quality [22], the self-reported questionnaires on difficulty sleeping at 2,400 m and 3,400 m did not differ between subjects with and without AMS (LLAMS ≥ 3, < 3), which might not have confounded the interpretation of data. In addition, between the two groups of subjects with and without AMS, the Δδ power of EEG at the FP1 and P3 electrodes at moderate attitude and ΔMPF_EEG_ and Δδ power at the P4 at high attitude showed significant differences. At moderate altitude, the Δδ power increase of EEG at the P4 electrode could be a predictor of the headache symptom of AMS before ascending to high altitude. In addition, Δδ power changes at the T4 and P4 electrodes and MPF_EEG_ from ground to high altitude (3,400 m) were correlated with LLAMS scores. These sequential spectral changes in EEG may be more sensitive than changes in commonly measured physiological parameters, such as SPO_2_, for the prediction of whether subjects will present AMS after ascending to high altitude.

### Changes of electroencephalography during exposure to a high altitude environment

While people are exposed to hypobaric hypoxic environments, neurological changes and brain modulation are also important and related to the symptoms of AMS. Evidence from animal studies at the cellular level has shown that during hypoxia, the hippocampus is particularly at risk through intracellular Ca^++^ and glutamate increase [23, 24]. Reports of neuropsychological changes during ascent to extreme altitudes underline the vulnerability of the nondominant right hemisphere. For instance, a decline in visual long-term memory may reflect a change in right temporal brain function due to hypobaric hypoxia [25, 26]. Transient high-altitude, neurological dysfunction, such as hallucinations, vestibular disruption, and dressing apraxia, may be due to neuronal dysfunction of the right inferior parietal and superior temporal cortices [27, 28]. According to the literature, cerebral blood flow (CBF) increases by 20% to 50% 12 to 24 hours after arrival at altitudes ranging between 3,475 and 4,559 m [29]. The subsequent rise in CBF is a compensatory mechanism that may partially restore oxygen delivery to the brain [30].

Feddersen et al. showed that mountaineers affected by AMS exhibited regional cerebral dysfunction before the onset of clinical symptoms of AMS [13], and the increase in delta activity in the nondominant right temporal lobe preceded the clinical symptoms of AMS. A regional increase in slow delta activity indicates nonspecific brain dysfunction, and such EEG changes are typically associated with disruptions of corticothalamic connections in the white matter, whereas vasogenic edema typically does not cause EEG slowing (delta activity) [31]. This change in cerebral activity could be due to a delayed response to hypoxia because patients suffering from AMS have a delayed increase in ventilatory response to it [10, 14]. An alteration of right temporal brain activity and an increase in δ frequency power of EEG are considered important early changes while people experience symptoms of AMS [13, 15]. In our data, we conducted a post-hoc power analysis, which revealed that the powers were between 0.05 and 0.17. This post-hoc analysis showed that one potential reason for not detecting the difference in EEG activity could be the lack of statistical power.

In our study, we recorded changes in EEG at six electrodes at different altitudes. Some statistical differences were observed in the power spectrum between the groups with and without AMS (LLAMS ≥ 3 and < 3). However, this trend is not clear and is not fixed at the same electrode. Considering that the symptom of headache is an important diagnostic criterion of AMS, we found that from both ground to 2,400 m and to 3,400 m, the δ power increase at the P4 electrode is significantly different between subjects with and without headache. In addition, compared with subjects with more symptoms of AMS (LLAMS ≥ 5) from the ground to 3,400 m, the Δδ frequency power at all six electrodes significantly increased. These findings may indicate that the Δδ frequency power increase in EEG is an important parameter of AMS development; however, this change could only be detected at higher altitudes (3,400 m) or in subjects with moderate AMS severity (LLAMS ≥ 5, with headache).

Because of its itinerary design and restrictions, the influence of exercise could not be evaluated in this study. All subjects were getting to 2,400 m from the ground by taking a bus and to 3,400 m from 2,400 m by trekking. On the first day, most subjects felt lazy and low-spirited after a bus tour of more than 7 hours to Lulin lodge. In contrast, some people felt excited, but others felt tired after a 5–6-h trek to Paiyun lodge (3,400 m) on the second day. The EEG parameters were recorded in the evening during these two days. Some changes in EEG may relate to exercise during daytime but not completely to the subjects’ exposure to hypobaric hypoxic environments.

### Prediction of AMS at moderate altitude before ascending to high altitude

Predicting the development of AMS while ascending to high altitudes remains an important issue in the literature. A previous study demonstrated that lower arterial oxygen saturation (SaO_2_) values in healthy mountaineers exposed to simulated normobaric or hypobaric hypoxia are susceptible to AMS [32]. Several studies have shown that of SpO_2_ measurements at rest (R-SpO_2_) and immediately after exercise (Ex-SpO_2_) are predictors of AMS [8, 9, 33]; however, these parameters, such as SpO_2_ and EEG, lack a large number of studies corroborating these results and cannot be replicated easily at different altitudes and in mountain environments. Currently, no physiological parameters detected at low altitude can be useful as sensitive measurements for the early prediction of subjects suffering from AMS while ascending to high altitudes.

Our study attempted to test whether the power spectrum of EEG could be a predictor of AMS before ascending to high altitude. In the parameters of EEG, the Δδ power increase at FP1 and P3 was significantly different in these two groups at moderate altitude (ground to 2,400 m); however, this change was not observed at high altitude (ground to 3,400 m). The consistency of Δδ power increasing at the P4 electrode both at moderate and high altitudes could only be observed between the two groups with and without headache. This difference in results compared with previous reports [13, 15] may be related to the different ascent rates of the study design and data recording at different altitudes. However, based on our results, changes in δ power at the P4 electrode from ground to moderate altitude could be a predictor of the headache symptom of AMS before ascending to high altitude.

### Limitations of the study

This study has some limitations. The number of study subjects was limited and separated in different seasons. We did not perform these subjects’ lung function tests at baseline. The subjects were predominantly female, and the use of medicines for the prevention or treatment of AMS was not restricted. The influence of exercise from ground to 2,400 m (by car) and 2,400 m to 3,400 m (by trekking) could not be calculated and compared. In addition, the quality of EEG signals was hard to control. The subjects easily sweated and could not wash their heads during the trekking itinerary. Complete signal data without noise were difficult to obtain, and it was necessary to select adequate sample segments manually on a computer after conducting the study. A more reliable, accurate, and easy method for obtaining good EEG signals is a key factor of EEG application in further AMS studies and popularization.

## Conclusions

At moderate altitude, the δ power increase of EEG at the P4 electrode could be a predictor of the headache symptom of AMS before ascending to high altitude. In addition, from ground to high altitude, larger increments in δ power were observed in subjects with moderate AMS severity (LLAMS ≥ 5). Differences in the MPF_EEG_ and δ activity of EEG at the T4 and P4 electrodes correlate with the LLAMS scores from ground to 3,400 m. The application of EEG, such as δ power frequency and MPF_EEG_, could be a predictive marker or diagnostic tool in AMS. Further studies including a larger number of subjects or a different ascent rate and higher altitude with subjects prone to AMS should be validated in future perspectives.

## Supporting information

S1 File (XLSX)
